# The stochastic dynamics of early epidemics: probability of establishment, initial growth rate, and infection cluster size at first detection

**DOI:** 10.1098/rsif.2021.0575

**Published:** 2021-11-17

**Authors:** Peter Czuppon, Emmanuel Schertzer, François Blanquart, Florence Débarre

**Affiliations:** ^1^ Institute of Ecology and Environmental Sciences of Paris (iEES-Paris, UMR 7618), Sorbonne Université, CNRS, UPEC, IRD, INRAE, Paris 75252, France; ^2^ Center for Interdisciplinary Research in Biology, CNRS, Collège de France, PSL Research University, Paris 75005, France; ^3^ Institute for Evolution and Biodiversity, University of Münster, Münster 48149, Germany; ^4^ Faculty of Mathematics, University of Vienna, Wien 1090, Austria; ^5^ Infection Antimicrobials Modelling Evolution, UMR 1137, INSERM, Université de Paris, Paris 75018, France

**Keywords:** early epidemic dynamics, establishment probability, renewal equation, mass testing, timing of detection, testing frequency

## Abstract

Emerging epidemics and local infection clusters are initially prone to stochastic effects that can substantially impact the early epidemic trajectory. While numerous studies are devoted to the deterministic regime of an established epidemic, mathematical descriptions of the initial phase of epidemic growth are comparatively rarer. Here, we review existing mathematical results on the size of the epidemic over time, and derive new results to elucidate the early dynamics of an infection cluster started by a single infected individual. We show that the initial growth of epidemics that eventually take off is accelerated by stochasticity. As an application, we compute the distribution of the first detection time of an infected individual in an infection cluster depending on testing effort, and estimate that the SARS-CoV-2 variant of concern Alpha detected in September 2020 first appeared in the UK early August 2020. We also compute a minimal testing frequency to detect clusters before they exceed a given threshold size. These results improve our theoretical understanding of early epidemics and will be useful for the study and control of local infectious disease clusters.

## 1. Introduction

The emergence and spread of infectious diseases pose an increasing threat in an ever more interconnected world. A quantitative understanding of epidemic dynamics is necessary to improve control measures. Deterministic models are a suitable tool to describe the epidemiological dynamics once a large number of individuals has been infected. During the early phase of an epidemic in a local infection cluster however, stochastic effects cannot be neglected. These stochastic effects are due to the initially low number of infected individuals, and to the inherent stochasticity of the transmission process. Understanding and quantifying these stochastic effects will help, for example, assess the risk of new infection clusters emerging or estimate the size of a cluster associated with a new variant when such a variant is detected.

The infectiousness of an individual may vary over the course of their infection because of within-host viral dynamics if the transmission rate is correlated with the viral load. We consider a generic stochastic model in which infectiousness is an arbitrary function of time since infection. This stochastic model is called a Crump–Mode–Jagers process [1–3]. When the number of infected individuals becomes large, this stochastic model can be approximated by a deterministic partial differential equation describing the distribution of the time since infection of the host population. This equation is known as the McKendrick–von Foerster partial differential equation [4–6].

Transmission timings are particularly influential during the early stages of the growth of an infection cluster, which is the focus of our work. It is therefore important to use biologically realistic distributions of transmission times [7], rather than assuming mathematically convenient but biologically unrealistic exponential distributions. A constant infectiousness over the duration of an individual’s infection leads to the predominantly used framework of ordinary differential equations (ODEs), while non-constant infectiousness can be captured by a partial differential equation. In addition to the added biological realism, a time-varying infectiousness of infected individuals can also properly capture the dynamical consequences of abrupt changes in transmission rate [6,8]. This is not possible with an ODE framework [9].

Here, we provide key results about the epidemic dynamics as described by the McKendrick–von Foerster equation. Stochasticity in transmission does not merely add noise to the dynamics, but also causes a systematic deviation from the deterministic description, which underestimates the initial growth of an establishing epidemic [10,11]. This is in contradiction to a common misconception that stochasticity generally slows down the initial epidemic growth rate. We quantify the deviation between the deterministic and observed stochastic growth rates by conditioning the individual-based process on survival. After initial stochastic effects, the process converges to exponential growth with an asymptotic growth rate, denoted *r*, derived from the reproduction number *R* and the transmission rate. The distribution of time since infection in the stationary regime is exponential with parameter *r*, the asymptotic growth rate.

The reviewed and newly derived results can inform public health-related questions: how many importations will eventually result in a local infection cluster? How large is a local cluster once a first case is detected? When did a new variant—like Alpha, first detected in the UK—arise? How large is the detection rate of infectious individuals by a single mass testing effort? How many daily tests need to be conducted to detect local clusters before they exceed a certain size? We show how our theoretical results provide quantitative answers to these questions.

## 2. Expected epidemic size

We study the epidemic size of a cluster initiated by a single infected individual. By ‘cluster’ we refer to the entire tree of infections initiated by a single infected individual. In particular, we do not spatially restrict a cluster, nor do we constrain the time period in which transmissions need to occur.

**Figure 1.**
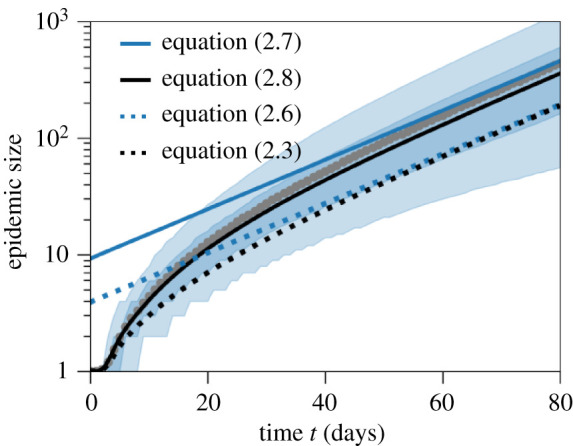
Cumulative number of infected individuals over time. The light and dark shaded regions show the 90% and 50% inter-quantile ranges obtained from 10 000 stochastic simulations that resulted in cluster establishment. Grey dots show the average of these simulations over time. The theoretical prediction (black solid line) is calculated from equation (2.8) with the adjusted transmission rate as computed in electronic supplementary material, §S5. The black dotted line shows the prediction obtained from equation (2.3), i.e. without conditioning on the epidemic to establish. The solid blue line is the epidemic size predicted by the asymptotic growth rate as stated in equation (2.7). The blue dotted line is the corresponding quantity without the stochastic adjustment (equation (2.6)). The effective reproduction number is set to *R* = 1.3, the number of secondary transmission events is Poisson-distributed, and the transmission density *μ*(*t*) is a gamma distribution with the parameters given in table 1.

Because some of our developments will also need them, we first recall results on *deterministic* epidemiological dynamics. We then develop new analytical results on the expected early growth and the expected number of infected individuals once a stationary regime has been reached. We illustrate with simulations the variability across stochastic trajectories (figure 1). As observed before [10,11], the expected growth rate during the early phase of cluster growth is greater than the long-term deterministic expectation, because clusters that do not die out are typically those that initially grow faster. We show how to account for this phenomenon in the mathematical description of the early phase and of the stationary regime.

In our stochastic simulations, we assume that the epidemic starts with a single infected individual at time *t* = 0. Each infected individual *i* is assigned a time since infection *a*_*i*_. The time since infection determines the infectiousness of an individual through time. The term ‘time since infection’ is also referred to as ‘age of infection’ in the mathematical literature. We decouple the transmission rate *τ*(*a*) into a mean number of secondary infections *R* and a transmission probability density over time *μ*(*a*). We then have

2.1 τ(a)=R×μ(a).

This equation holds because $\int_{0}^{\infty }\mu (a)da=1$, so that indeed the average number of secondary infections is given by *R*. This decoupling allows us, in a relatively simple way, to study different offspring distributions for *R*, while leaving the transmission density *μ*(*a*) unchanged.

For illustration, we assume that the distribution of transmission times follows a gamma distribution, but any distribution would be possible. In particular, a constant transmission rate (uniform distribution) would result in an exponential distribution of the transmission times (i.e. the memory-less distribution), which would reduce this general model into an ODE.

### 2.1. Previous results on deterministic dynamics: renewal equation, growth rate and time-since-infection distribution

Throughout our analysis, we assume that the fraction of susceptible individuals is sufficiently large compared to the number of individuals infected in the early epidemic, so that it remains approximately constant. The overall rate at which new infections occur at time *t*, denoted by *i*(*t*), in the deterministic regime is described by the following renewal equation [12]:

2.2 $$i(t)=\tau (t)+\int_{0}^{t}\tau (a)i(t-a)\;da,$$

where *τ*(*a*) is the transmission rate of an individual with time since infection *a*. The first term *τ*(*t*) reflects the new infections by the first infected individual at time *t*. The integral in equation (2.2) is the continuous version of the sum over the number of new infections caused by individuals with time since infection *a* (term *i*(*t* − *a*) d*a*), which happens at rate *τ*(*a*). Intuitively, one can think about *i*(*t*)d*t* being the incidence at time *t*, i.e. the number of newly infected individuals in the small time interval [*t*, *t* + d*t*].

The cumulative number of infected individuals, i.e. the total epidemic size, which we denote by *I*(*t*), is then given by

2.3 $$I(t)=1+\int_{0}^{t}i(s)\;ds=1+\int_{0}^{t}I(t-a)\tau (a)\;da,$$

with *I*(0) = 1 (mathematical details are given in the electronic supplementary material, §S4). For simplicity, we do not consider recovery of infected individuals. However, individuals will of course stop transmitting when the time since infection is such that the transmission rate *τ*(*a*) becomes very small.

The epidemic size *I*(*t*) will, for large times *t*, grow exponentially if *R* > 1. Formally, the asymptotic exponential growth rate *r* is obtained by solving the classical Euler–Lotka equation [12,13]:

2.4 $$1=\int_{0}^{\infty }e^{-rt}\tau (t)\;dt\;\Leftrightarrow \;\frac{1}{R}=\int_{0}^{\infty }e^{-rt}\mu (t)\;dt,$$

where *r* is also called the Malthusian parameter of the supercritical branching process [14]. In the case where *μ*(*t*) is given as the density of a gamma distribution with shape parameter *α* and scale parameter *β*, the exponential growth rate *r* is

2.5 $$r=\frac{R^{1/\alpha }-1}{\beta }.$$

Convergence speed from the initial condition towards the asymptotic growth rate *r* is determined by the average number of secondary infections *R* and the transmission probability density *μ*. Intuitively, the faster a large number of infected individuals is reached (high *R* and/or small average transmission time), the faster is convergence towards the stationary growth regime.

Furthermore, it is possible to derive an explicit expression for the number of infected individuals over time, once asymptotic growth is reached. It follows from results of supercritical general branching processes and renewal theory [14] that the expected cumulative epidemic size is, for asymptotically large times *t*, given by

2.6 $$I(t)=I(0)\frac{e^{rt}}{rR\int_{0}^{\infty }e^{-rs}s\mu (s)\;ds}.$$

The integral in the denominator is the mean generation time of the Malthusian process [13,15]. This is the mean time between the infection of the infecting individual and the time of infection of a randomly chosen secondary infection event. If the transmission density *μ*(*s*) was constant, the integral would be 1/(*rR*) and the epidemic size would be the solution of a constant infection process without depletion of susceptibles: *I*(*t*) = *I*(0)e^*rt*^.

For an uncontrolled COVID-19 epidemic (we set *R* = 2.9, estimated for the French epidemic in Spring 2020 [16]), we obtain *r* ≈ 0.18 per day, which corresponds to a doubling time of about 4 days. When interventions are in place (e.g. *R* = 1.3), then the Malthusian parameter is *r* ≈ 0.048 per day, which corresponds to a doubling time of 14 days.

Under exponential growth, the distribution of the ages of infection in the population is given by an exponential distribution with parameter *r*, the exponential growth rate [14]. Intuitively, in an exponentially growing population, the number of individuals who were infected *a* days ago is e^*r*^ times greater than the number of individuals who were infected *a* + 1 days ago. The exponential distribution also implies that for a large growth rate *r*, a large proportion of the cumulative number of infections will be very recent. For example, with *R* = 2.9, 30% of the total cumulative number of infections occurred within the last 2 days.

We now turn to the stochastic simulations and show how systematic deviations from the deterministic regime can be understood and mathematically described. We first give a stochastic correction for the asymptotic growth rate and then apply a similar idea to the general epidemic size process over time.

### 2.2. Asymptotic growth rate and epidemic size in the stochastic epidemic model

For large enough times after the initially infected individual started the local cluster, the epidemic grows exponentially at the rate predicted by the Euler–Lotka equation (equation (2.4)). However, the expected cumulative epidemic size derived for the deterministic case (equation (2.6)) includes epidemics that eventually die out. Since we are only interested in epidemic clusters that eventually result in a large epidemic outbreak, we rescale the initial epidemic size by dividing by the survival probability *p*_surv_:

2.7 $$I_{\mathrm{surv}}(t)=\frac{I(t)}{p_{\mathrm{surv}}}=\frac{I(0)}{p_{\mathrm{surv}}}\frac{e^{rt}}{rR\int_{0}^{\infty }e^{-rs}s\mu (s)\;ds}.$$

This rescaling reflects conditioning of the epidemic process on survival (figure 1). Formally, the correction of the asymptotic limit in equation (2.7) is derived from a convergence result of a general branching process (electronic supplementary material, §S3). The survival probability is *p*_surv_ = 1 − *p*_ext_, where the probability of extinction *p*_ext_ is numerically computed as the fixed point of the probability generating function of the distribution of secondary infections. In other words, the probability of extinction is equal to the probability that the initial infected individual does not produce any secondary infection, plus the probability that it produces one secondary infection which goes extinct (*p*_ext_), plus the probability that it produces two secondary infections which both go extinct ($p_{\mathrm{ext}}^{2}$), and so on; this intuition is outlined in electronic supplementary material, §S1.

### 2.3. Initial stochastic growth of an epidemic

The initial growth rate of an epidemic that does not become extinct is initially steeper than its final asymptotic growth rate [10,11] (compare the initial slope of the mean of stochastic simulations with the asymptotic growth for large times; grey dots versus blue solid line in figure 1). This is due to the inherent stochasticity of the transmission process, which strongly affects the dynamics when there are only a small number of infected individuals. Clusters that escape extinctions are typically those that by chance benefited from a larger initial growth than the long-term expectation. This also means that deterministic models tend to underestimate epidemic sizes early on, or, if parameters are inferred from data, overestimate epidemic parameters such as the true basic reproduction number *R*_0_, as for example observed in [17].

To account for this initial stochastic phase, one can alter the individual-based dynamics by conditioning the stochastic process on the survival of the epidemic. A similar procedure has been employed in [11]. This conditioning results in an adjustment of the transmission rate *τ*, which we denote by $\tilde{\tau }$. Formally, this adjustment is only justified for the stochastic process by Doob’s h-transform [18] (details in electronic supplementary material, §S5). In the large population size limit, we then approximate the adjusted transmission rate by the continuous analogue of the adjusted transmission rate of the stochastic process. This approximation, while mathematically not fully justified, is a natural analogy of the conditioning of the asymptotic epidemic size in equation (2.7). The mean epidemic size of the adjusted process is then given by

2.8 $$\tilde{I}(t)=1+\int_{0}^{t}\tilde{i}(s)\;ds,$$

where $\tilde{i}(s)\;ds$ is the incidence in the time interval [*s*, *s* + d*s*) under the adjusted process. The rate of new infections $\tilde{i}(t)$ in the conditioned process now depends nonlinearly on the history of the epidemic and therefore does not satisfy a renewal equation as in equation (2.2), but a delay differential equation

2.9 $$\tilde{i}(t)=F(\tilde{i}(s);s\in [0,t]).$$

The function *F* is explicitly computed in electronic supplementary material, §S5 (equation (S37)). In short, the conditioning on survival of the epidemic results in an adjustment of the transmission rate *τ* by a factor that varies over time. This adjustment factor reflects the survival probability of the epidemic at a certain time and depends on the size and the age structure of the epidemic over time. The adjustment factor is largest at time *t* = 0, where it equals (1 + *p*_ext_). Over time, the adjustment factor decreases and asymptotically approaches 1 for a large epidemic size, where the probability of extinction becomes negligible, i.e. for large times $\tilde{\tau }=\tau$.

In figure 1, we plot both the adjusted and non-adjusted versions of the mean epidemic size (equations (2.3) and (2.8)). As mentioned above, the non-adjusted formula (black dotted line) underestimates the mean epidemic sizes as obtained from 10 000 stochastic simulations (grey dots). By contrast, conditioning the transmission density on survival (black solid line) predicts the mean epidemic size over time reasonably well, and also equilibrates approximately at the correct level. Overall, there is large variation in the epidemic sizes between different trajectories, as shown by the broad light shaded region corresponding to the 90% inter-quantile range of the simulated trajectories. To model the number of secondary infections, we have used the Poisson distribution in the figure because the adjustment of the transmission rate does not result in explicit expressions if a negative binomial distribution is used. Cumulative epidemic sizes in case the number of secondary infections is distributed according to a negative binomial or geometric distribution show more variation due to the larger variance in the number of secondary infections (electronic supplementary material, figure S2 in §S6).

## 3. Applications

We now apply the theoretical results obtained above. First, we use the approximation of the epidemic size (equation (2.8)) to estimate the probability distribution of the emergence time of the Alpha variant, first detected in the UK in September 2020. The distribution of the emergence time also provides insight into the probability distribution of the size of the cluster when the variant was first sampled. As a second application, we estimate the minimal testing frequency necessary to detect new emerging clusters before they exceed a certain size (on average). This prediction is especially relevant when the number of infected individuals is rare.

### 3.1. Distribution of the first detection time and cluster size at detection, and application to the origin of the Alpha variant

The Alpha variant initially consisted only of the B.1.1.7 lineage. This lineage was first detected in the UK from a sample that was collected on 20 September 2020 [19] and has rapidly become a major variant of concern due to its increased transmissibility [20] and pathogenicity [21]. Here, we develop a method to estimate the date of the first infection of an individual with the Alpha variant and the distribution of the size of the Alpha cluster on the day when the sample was taken in September, based on the dynamics of the epidemic size of a local cluster.

Our analysis requires the effective reproduction number, estimated to be *R* = 1.5 for the Alpha variant in November 2020 in the UK [20], and the probability for a sample taken in the UK to be sequenced, which was around 4.2% in October 2020 [22]. We will use this value in our analysis, keeping in mind that this might be an underestimate because the number of cases were lower in September so the percentage of samples that could have been sequenced was potentially higher. Since only reported cases can be sampled, we additionally account for underreporting of cases. We assume that around 25% of all infections are detected [23]. Lastly, we need to define a distribution for the time that passes between infection and sampling of an infectious individual. We assume that the time from infection to sampling is a gamma distributed random variable (but any distribution would work) with a mean of 7 days and a standard deviation of 2 days. The parameter values (table 1) are chosen such that they give a probability of sampling and sequencing an infected individual up until 3 days of their infection that is less than 1%, and a probability of sampling an infected individual after 10 days of their infection that is less than 10%. All parameters are summarized in table 1.

**Table 1.** Probability distributions and parameter values used in the case study of the Alpha variant.

interpretation	distribution	parameters	reference
mean number of secondary infections	Poisson	*R* = 1.5	[20]
time of secondary infection	gamma (density: *μ*(*t*))	shape: 6.6, scale: 0.833 (mean: 5.5 days)	[24]
time from infection to sampling	gamma (density: *f*_sampling_(*t*))	shape: 12, scale: 7/12 (mean: 7 days)	—
sequencing probability	Bernoulli	*p*_sequencing_ = 0.042	[22]
sampling probability	Bernoulli	*p*_sampling_ = 0.25 × *p*_sequencing_	[23]

#### 3.1.1. Distribution of the first detection time

To estimate the time of the first detection of an individual infected by the Alpha variant, we combine the sampling probability distribution *f*_sampling_ with the expected epidemic size at time *t*, given by the adjusted version of the epidemic size in equation (2.8), and the number of infections until the first infected in the cluster is sampled and sequenced, which happens with probability *p*_sampling_ per infected individual. For readability, we refer to this first infected individual that is sampled and sequenced by case X and only write sampling when in fact we mean sampling and sequencing. The number of infection events until case X is infected, including case X, is denoted *N*_inf_. It is a geometrically distributed number with probability *p*_sampling_. Note that if we were interested in the *j*th sampling event, the number of infected individuals until the *j*th sampling event would be distributed according to a negative binomial distribution with ‘success’ probability *p*_sampling_ and dispersion *κ* = *j*.

We combine the distribution of *N*_inf_ with the *deterministic* time needed for the infected population to reach *N*_inf_ individuals (conditioned on non-extinction of this epidemic cluster as computed in equation (2.8)). We also refer to this time as *hitting time* and denote it by $t_{N_{\inf}}^{\det}$. To this, we add the time from infection of case X to their sampling. Denoting by *T*_sampling_ the random variable corresponding to the time of first detection and sampling, its probability density is given by

3.1 $$\begin{array}{rl} h_{\mathrm{sampling}}(t) & \;:=\lim_{dt\to 0}P(T_{\mathrm{sampling}}\in (t-dt,t+dt))\\ & \approx \sum_{i=1}^{\infty }P(N_{\inf}=i)\;f_{\mathrm{sampling}}(t-t_{i}^{\det})\\ & =\sum_{i=1}^{\infty }p_{\mathrm{sampling}}{\left(1-p_{\mathrm{sampling}}\right)}^{i-1}f_{\mathrm{sampling}}(t-t_{i}^{\det}), \end{array}$$

where *f*_sampling_(*s*) denotes the probability density of the time from infection to sampling evaluated at time *s* (table 1). We emphasize that the density of the first sampling time *h*_sampling_(*t*) is an approximation, because it is based on the mean epidemic size and not the whole distribution of the epidemic size. The mean epidemic size directly provides the deterministic hitting time $t_{i}^{\det}$, neglecting the whole distribution of the epidemic size.

**Figure 2.**
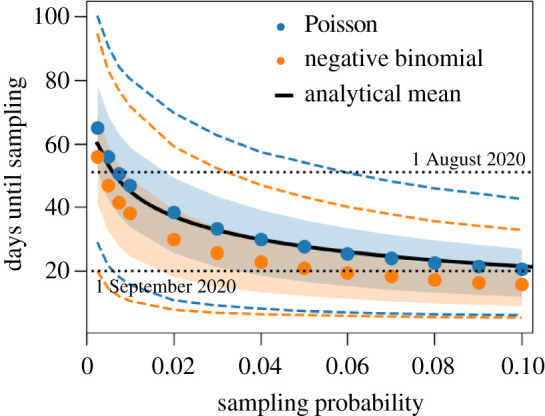
The date of emergence of the first infection with the Alpha variant in the UK when varying the sampling probability. The shaded regions and dashed lines show the 50% and 90% inter-quantile ranges obtained from 10 000 stochastic simulations that resulted in cluster establishment; blue for the secondary infections being Poisson distributed, orange for a negative binomial distribution. Dots represent the means of these simulations when varying the sampling probability. The effective reproduction number is set to *R* = 1.5, the dispersion parameter is *κ* = 0.57 [16], and the transmission density *μ*(*t*) and the waiting time between infection and sampling (*f*_sampling_) are gamma distributions with parameters as stated in table 1. The theoretical mean (black solid line) of the first sampling time is calculated from equation (3.1), which only applies to the Poisson case.

With our COVID-19-specific parameter set given in table 1, we find that the mean time between the first infection of an individual with the Alpha variant and sampling of case X is around 46 days, indicating that the strain was present in the UK on 4 August 2020—yet, the variance is quite large for this distribution: the standard deviation is 19.5 days. The emergence date of the Alpha variant strongly depends on the sampling probability: smaller sampling probabilities result in earlier possible emergence dates than larger probabilities (figure 2). The distribution of secondary cases also impacts the timing: if the number of secondary infections is distributed as a negative binomial distribution, the date of emergence shifts closer to the date of sampling of case X. This effect is secondary though, compared to the impact of the sampling probability (figure 2).

**Figure 3.**
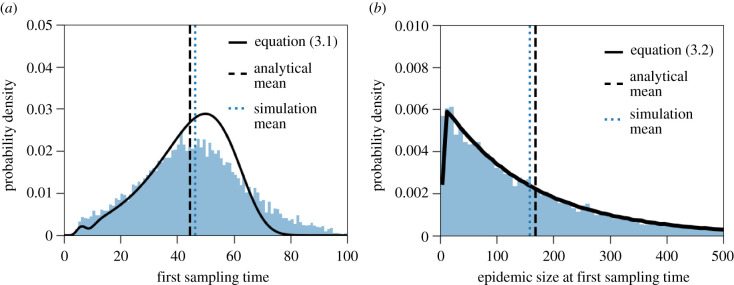
Distribution of the first sampling time and the cluster size at that time, parameterized to the case of the Alpha variant. The histograms are obtained from 10 000 stochastic simulations and represent (*a*) the first sampling time of an infected individual with the Alpha variant, measured since the first infection of an individual with the Alpha variant (in days), and (*b*) the cluster size at this first sampling time. The theoretical predictions (black solid lines) are given by equations (3.1) and (3.2). The parameters and distributions used in the stochastic simulations are given in table 1.

In general, we find that the theoretical prediction of the probability distribution of the first sampling time captures the shape of the empirical distribution from the stochastic simulation results (figure 3*a*). Note that this implies that most of the variability in time does not come from stochasticity in epidemic size, but from the variability emerging from the random sampling of infected individuals (*p*_sampling_) and the variability in the time from infection to sampling of infected individuals (*f*_sampling_). Biologically, the variability in the time from infection to sampling arises from inter-individual variability in viral dynamics, symptom development, test seeking behaviour, etc. We find the largest discrepancy between theory and simulations at large first sampling times, i.e. we underestimate the right tail of the first sampling time distribution. This difference arises because our theoretical approximation does not take into account variability in the epidemic size process. Figure 1 shows a large variation in the number of infected individuals over time between different stochastic trajectories. Most notably, there are several trajectories that remain at low cumulative epidemic sizes for a relatively long time. These trajectories are responsible for the long right tail of the sampling time distribution in figure 3*a*.

#### 3.1.2. Cluster size at the first detection time

Next, we use this distribution of the first sampling time to infer the size of the epidemic cluster at that time. Therefore, we combine the adjusted epidemic size in equation (2.8) with equation (3.1) and obtain the following probability mass function for the size of the cluster at the sampling time of case X:

3.2 $$P(I(T_{\mathrm{sampling}})=k)=\int_{0}^{\infty }h_{\mathrm{sampling}}(t)1_{\left\{\tilde{I}(t)\in [k-1/2,k+1/2)\right\}}dt,$$

where *h*_sampling_ is the probability that the first sampling time lies in the interval [*t*, *t* + d*t*), given in equation (3.1).

This estimate of the epidemic size distribution approximates the simulated data reasonably well (figure 3*b*). The only notable difference occurs for very low epidemic sizes, where the epidemic size at the first sampling time ranges from 0 to 8 (bin size is set to 8—the smallest bin size that produces a continuous theoretical prediction), as can be seen in the histogram in figure 3*b*. The mean size of the cluster with the Alpha variant at the first sampling time (obtained from stochastic simulations) consists of 159 individuals, yet again with a large standard deviation of 158 individuals. For example, the 95-percentile of the simulations predicts a cluster size of 476 infected individuals with the Alpha variant by the time of the first sampling of the variant.

### 3.2. Minimal testing frequency to detect clusters of a given size

A single mass testing effort only results in a detection rate of between 25 and 48% of potentially infectious individuals, depending on the test used (rapid test or polymerase chain reaction) and the exponential growth rate *r* corresponding to reproduction numbers *R* between 1.3 and 3 (details in electronic supplementary material, §S7). Therefore, we now ask whether repeated random testing in the population is a more feasible strategy to contain an infection cluster. Specifically, how often should we randomly test the population to detect a cluster before it exceeds a certain size? As a numerical example, we will use a threshold cluster size of 30 infected individuals. We assume that testing is applied population-wide at random, independently of the infection state of an individual. The probability to test positive depends on the time since infection of an individual [25–27]. We denote the probability to test positive by a rapid test if the infected individual has been infected *a* days ago by *Q*(*a*) (electronic supplementary material, figure S3 in §S7).

If a fraction *f* of the population is tested every day, the detection probability of an infected individual is approximately given by

3.3 $$p_{\mathrm{detect}}=1-\prod_{a=1}^{\infty }(1-fQ(a))\approx f\sum_{a=1}^{\infty }Q(a).$$

The term (1 − *f Q*(*a*)) is the probability that an infected individual is not detected at their time since infection *a*. Hence, the product is the probability that an individual is never detected over the course of their infection. The probability of detection is one minus this product. The approximation is valid when it is very unlikely that the same individual is tested more than once during the period when there is a high chance to detect their infection.

To determine the testing frequency above which the expected cluster size is smaller than 30 infected individuals, we repeat the steps from the previous sections: first, we determine the first detection time and then translate this result to the average cluster size at detection. Since our analytical result tends to overestimate the cluster size at detection (electronic supplementary material, figure S4 in §S8), this analytical procedure will provide an upper bound for the true testing frequency required to detect clusters of a certain size. In our numerical example with *R* = 1.1, this procedure results in a testing frequency of 0.13% for a threshold cluster size of 30 infected individuals.

Importantly, increasing the testing frequency when it is still low offers large benefits in terms of cluster size at detection because the epidemic size at detection reflects the exponential growth of the epidemic: it decreases exponentially with increasing testing frequency (electronic supplementary material, figure S4 in §S8).

## 4. Discussion

We have collected key equations and derived new results to account for stochasticity during the early phase of epidemic trajectories. Explicitly taking into account stochastic effects during the early phase of an epidemic allowed us to compute a good description of the mean epidemic size for all times (equation (2.8)). Importantly, our result captures the increased initial growth rate of surviving epidemics when compared to the asymptotic growth rate (figure 1). This is a known effect [10,11], yet cannot be captured by deterministic epidemiological models. One important consequence of this theoretical underestimation of classically used models is that parameter inference during the early phase of an epidemic of, for example, the basic reproduction number, will result in an overestimation of the true value [17]. We provide a new mathematical description of the expected epidemic size over time that could be used in statistical inference during the early phase of emerging epidemics.

As a first application, we analytically derived the probability distribution of the first detection time of an epidemic cluster. While in principle applicable to any type of detection event, as for instance the first death or the first hospitalization event, we have focused on dating the emergence of the Alpha variant that was first sampled in the UK on 20 September 2020. Our analysis is appropriate for clusters that descend from a single infected individual, and as long as population immunity is low enough for the supply of susceptible individuals to be unlimited. The Alpha variant was first detected in England in September 2020 and likely emerged there once, so our analysis can be applied to it. It would not apply, for example, to the Delta variant in the UK, unless the cluster linked to the first importation of the variant could be identified—and so the date of importation could be estimated. On average, we find that the Alpha cluster was started 46 days before its detection, which means that the variant was likely present in the UK on 4 August 2020. Usually, phylogenetic methods are used to date the evolutionary history of mutations [28]. In this particular case, a phylogenetic approach is difficult because of the large divergence between Alpha and non-Alpha variants sampled at a similar time [19]. Indeed, we did not find a published estimate of the date of emergence of the Alpha variant based on a phylogenetic analysis. In an attempt to date the origin of SARS-CoV-2, a combination of phylogenetic and epidemiological methods has been used to obtain a more complete picture of the very early dynamics of the COVID-19 epidemic [29]. Our new description for early epidemic growth provides a formal non-spatial description of the individual-based simulations that were used in [29] to date the very first COVID-19 case.

We additionally derived an analytical approximation for the probability distribution of the epidemic size at the first detection event. In contrast to a previous numerical estimate of the cluster size at the first disease-caused death, which relies solely on the waiting time distribution until detection, e.g. the distribution from infection to death [30], we consider the whole epidemic trajectory of the cluster, i.e. from the first infected individual to the day of detection. The previously proposed method [30] inevitably results in an overestimate of the actual epidemic size. Previous research has also shown that if the probability of detection since infection were constant over time, which is not the case in our setting, the cluster size at detection would be geometrically distributed [31,32]. Whether the distribution of cluster sizes at detection is a geometric distribution if the detection process is not constant in time, is an open question. In our specific dataset, this seems to be the case (figure 3*b*).

We also applied our results to the evaluation of testing strategies. Currently (May 2021), aside from vaccination campaigns, frequent testing is seen as a possible solution to relax COVID-19-related restrictions in the short term. Our modelling approach gives an estimation of the minimal testing frequency per day to detect epidemic clusters of a certain size, for example small enough for manual contact tracing to be feasible. The minimal testing frequency depends on the test that is employed. In our numerical example, we have used the detection probability estimated for rapid tests, which were collected during the early phase of the epidemic in the UK in 2020 [27]. Since then, tests have improved so that our estimation of the minimal testing frequency is very likely an overestimate. We find that for a cluster size to be below 30 infected individuals (on average), each day around 0.13% of a total population would need to be randomly selected for testing, i.e. independently of the individual’s infection status. Pooled sample testing strategies could be a solution to reduce the number of testing kits needed, and is a particularly reasonable option when the prevalence of infected individuals in a community is close to zero [33].

Additionally, we estimated the fraction of cases that can be detected during a single mass testing effort, as has been for example conducted in Slovakia in autumn 2020 [34]. We find that with either a rapid test or a polymerase chain reaction test and with a reproduction number between *R* = 1.3 and *R* = 2.9, the detection rate of infectious individuals is between 26 and 48% (electronic supplementary material, §S7). During the mass testing effort, a certain fraction of undetected individuals is still in the latent phase (0–3 days post-infection) and will become infectious after the mass testing event. Similar observations have also been made by using a deterministic SEIR-model [35]. This indicates that only isolating positively detected individuals would be insufficient to contain the epidemic and that mass testing would need to be repeated to efficiently control the epidemic.

In conclusion, we have summarized existing theoretical results describing the early, stochastic dynamics of an epidemic, and developed new results on the mean epidemic size trajectory. We combined the establishment probability with the deterministic McKendrick–von Foerster equation to obtain a precise description of the expected epidemic size of an establishing epidemic over time. As an application, we approximated the probability distribution for the timing of a first infected individual in an epidemic cluster. This distribution can be used to estimate, for example, the emergence of new variants of a pathogen, like the Alpha variant. In addition, we derived the minimal testing frequency to detect clusters below a certain size. These applications are relevant from a public health perspective and could be used to guide the policy to contain and fight any infectious disease.

## Data accessibility

The C++ codes, data files and Python scripts used to generate the figures are available from the Dryad Digital Repository: https://doi.org/10.5061/dryad.7m0cfxpvv [36].
